# Codon Usage Bias and Host Adaptation of Canine Circovirus Identified from Various Hosts

**DOI:** 10.3390/ani16213494

**Published:** 2026-11-04

**Authors:** Ruiqi Jiao, Xin Xu, Jun Ji, Yanhua Wang, Lunguang Yao

**Affiliations:** 1 Henan Provincial Engineering Laboratory of Insects Bio-Reactor, Henan Provincial Engineering and Technology Center of Health Products for Livestock and Poultry, Henan Provincial Engineering and Technology, Animal Disease Diagnosis and Integrated Control, Nanyang Normal University, Nanyang 473061, China; 2 Henan Quality Monitoring Inspection and Research Institute of Fiber and Textile Products, Zhengzhou 450007, China

**Keywords:** dog circovirus, cross-species transmission, codon usage bias, adaptation, correspondence analysis

## Abstract

Canine circovirus (CCV) infection could cause immunosuppression in dogs, predisposing them to secondary complications. The virus also shows notable cross-species transmission potential. To investigate codon usage bias (CUB) and host adaptation, 102 CCV sequences from various hosts, including dogs, wolves, foxes, cats, and badgers, were analyzed. The mean GC% of CCV’s complete coding sequences was 50.31% (σ = 0.47), indicating no significant compositional bias. Furthermore, the replication protein (Rep) gene of CCV exhibited significant overrepresentation of T-ending codons and strongly avoided A-ending codons, whereas the capsid protein (Cap) gene displayed a clear codon bias toward A-ending codons and avoidance of G- or C-ending codons. Effective number of codons, parity rule 2, and neutrality analyses collectively indicated that natural selection acted as the primary evolutionary force governing the codon usage bias of CCV. Correspondence analysis (CoA) and correlation analysis demonstrated that other factors such as nucleotide composition and translational preferences could also affect CCV’s CUB. However, geography and genotype should not be ignored. The host adaptation analysis suggests that foxes are the most suitable hosts for CCV. This study provides a foundation for understanding CCV’s host adaptation mechanisms.

## Simple Summary

Canine circovirus (CCV) can cause immunosuppression in dogs. Our research has found that CCV codon usage was shaped by natural selection, with Rep preferring T-ending codons and Cap preferring A-ending codons. Based on codon usage bias, we speculate that foxes are the most suitable host.

## 1. Introduction

Canine circovirus (CCV) is a small (15–16 nm) virus lacking a viral envelope. It is a recently identified member of the genus Circovirus of the family *Circoviridae* [1,2,3]. CCV has a single-stranded circular DNA (ssDNA) genome spanning about 2063–2064 nucleotides and harboring two major open reading frames (ORF1 and ORF2) that code for the replication-associated protein (Rep) and capsid protein (Cap) [4,5]. Recent research has reported that ORF3, potentially encoding an additional replication-associated protein, is located on the antisense strand overlapping ORF1 (470~889) [6]. CCV infection has been documented to cause canine vasculitis and hemorrhagic gastroenteritis, accompanied by lymphocyte depletion and immunosuppression, which could result in secondary infections, posing a significant threat to the lives of dogs [2,7]. Following its initial detection in 2011, CCV has been found worldwide in at least five different host species: dogs (*Canis familiaris*), wolves (*Canis lupus*), foxes (*Vulpes vulpes*), cats (*Felis catus*) and badgers (*Meles meles*) [4,8,9,10].

During translation, codons specify amino acids, thereby mediating the transfer of genetic information from mRNA to amino acids [11]. For protein synthesis, codons transmit information in triplet form, with each amino acid corresponding to one to six synonymous codons [12]. Throughout its evolutionary history, CCV has developed biased synonymous codon usage patterns (CUP) across various hosts, shaped by the combined effects of natural selection, mutational pressure, and compositional constraints [13]. The preferential use of specific synonymous codons by a species or gene, termed codon usage bias (CUB), represents a basic characteristic of molecular evolution. CUB is widespread in nature, primarily influenced by natural selection and mutation pressure [14]. Viruses, owing to their obligate intracellular parasitism, entirely depend on the host cell for replication and translation activities. Therefore, to ensure rapid replication and evade the host’s immune surveillance, viruses must efficiently utilize the host cell’s intracellular environment to enhance translation efficiency and accuracy [15]. Over the course of evolution, viruses have developed various strategies to optimize the translation process within the host cell. These strategies include accelerating the translation process through rolling circle replication, prioritizing the use of the host’s ribosomes and tRNA pool to rapidly synthesize viral proteins, and altering codon usage preferences to adapt to the host cell’s translation machinery [5,16].

With the widespread dissemination of CCV and its expanding host range, it is imperative to understand the adaptive mechanisms of CCV. This study elucidates CCV host adaptation from the perspective of codon usage and provides a basis for future studies on the evolution and cross-species transmission of CCV.

## 2. Materials and Methods

### 2.1. Dataset Collection

Here, 102 full-length CCV genomes were obtained from the National Center for Biotechnology Information (NCBI), encompassing all CCV strains identified in currently known hosts, including dogs (66 sequences), wolves (16 sequences), foxes (15 sequences), cats (4 sequences) and badgers (1 sequence) (Supplementary Table S1). The coding regions of all 102 sequences were extracted; the termination codon of the Rep gene and the start codon of the Cap gene were removed, and the coding regions were joined end-to-end in coding order to form the complete coding sequences (CCDs). The 102 CCDs were classified based on their respective hosts (information obtained from GenBank annotations) and genotypes (genotyping information derived from the previous study) [17].

### 2.2. Base Profile Analysis

To systematically understand the nucleotide composition of CCVs, nucleotide abundances (A, T, C, G), nucleotide frequencies at synonymous sites (A3s, T3s, C3s, G3s) of CCV, GC composition in the third position of the degenerate codons (GC3s) and the effective number of codons (ENC) were all determined using the default parameters of CodonW (http://codonw.sourceforge.net/) and the CAI calculator (http://genomes.urv.es/CAIcal) (accessed on 13 July 2026).

### 2.3. Relative Synonymous Codon Usage Analysis of CCV

Relative synonymous codon usage (RSCU) is an index for evaluating the codon usage characteristics of different amino acids. MEGA (version 11) was used to calculate the RSCU of CCV’s ORF1, ORF2, and CCDs with reference to the hosts (dogs, wolves, foxes, cats, and badgers). The ATG (Met) and TGG (Trp) codons, together with the three stop codons TAA, TAG, and TGA, were excluded from the analysis [18,19].

In RSCU analysis, values > 1.0 indicate that synonymous codons were used more frequently than would be expected if all synonymous codons were used equally, values < 1.0 indicate less frequent use, and a value of 1.0 reflects neutral selection (usage equal to expectation). Moreover, RSCU values above 1.6 or below 0.6 signify overrepresentation or underrepresentation of the codon, respectively. The fact that these codons show the highest RSCU values in both the virus and its host may indicate a shared codon preference [20,21].

### 2.4. Codon Adaptation Index Analysis

The codon adaptation index (CAI), ranging from 0 to 1, reflects the degree of consistency between viral CUP and that of the infected host [12]. A greater CAI value reflects better viral adaptation to the host [11]. In this research, the CAI calculator online tool was used to calculate the CAI of CCV for different hosts, including dogs (*Canis familiaris*), wolves (*Canis lupus*), foxes (*Vulpes vulpes*), cats (*Felis catus*), and badgers (*Meles meles*) [22]. Reference codon usage datasets of each host were sourced from the database (https://www.kazusa.or.jp/codon/) (accessed on 29 July 2026) [23].

The relative codon deoptimization index (RCDI), an index that denotes the similarity between gene and host codon usage, was also calculated using the CAI calculator with the default parameters [24]. An RCDI value of 1 indicates perfect adaptation between viral codon usage and host preference. RCDI values > 1 reflect suboptimal adaptation, potentially leading to reduced replication efficiency or latency establishment, whereas values < 1 suggest enhanced host-specific adaptation. Based on RCDI, adaptability to the host can be predicted [25].

To evaluate the potential influence of host CUPs on viruses, the similarity index (SiD) [26] was calculated from RSCU values for the 59 synonymous codons in viral coding sequences relative to those for the same codons in the host genome, as previously described [3]. Generally, higher SiD values suggest a greater host influence on viral CUP. Low SiD indicates that viral and host codon usage are more similar, potentially suggesting a stronger host influence on viral codon usage, whereas high SiD indicates greater differences and lower similarity between viral and host codon usage [27,28,29].

### 2.5. Effect of Natural Selection and Mutation Pressure on the Codon Usage Bias

The effective number of codons (ENC) was analyzed to estimate the degree of CUB in CCV. ENC was plotted on the y-axis against GC3s on the x-axis. The expected curve shows the ENC scores at different GC3 contents under the exclusive influence of mutational pressure. Using the conventional threshold of 35, values below this cutoff indicate strong codon usage bias, while values above suggest more uniform codon usage with weaker bias [12]. The expected curve is defined by the following equation, where ‘s’ denotes the GC3s value [30].

$$ENC\;expected=2+s+(\frac{29}{s^{2}+{\left(1-s\right)}^{2}})$$

Using the groups of fourfold degenerate codons (alanine, glycine, proline, threonine, valine, arginine [CGA, CGU, CGG, and CGC], leucine [CUA, CUU, CUG, and CUC], and serine [UCA, UCU, UCG, and UCC]), parity rule 2 (PR2) analysis was performed. The G3/(G3 + C3) ratio was plotted against the A3/(A3 + T3) ratio to assess the effects of natural selection and mutational pressure [31].

Neutrality curve analysis was also performed to assess the relative contributions of mutation and selection to codon usage. The GC3 content (%) was plotted on the x-axis, and the mean GC content (%) at codon positions 1 and 2 (GC12) was plotted on the y-axis. Regression analysis was performed, with the regression line’s gradient representing the proportional contribution of mutational pressure to CUB [31,32].

### 2.6. Correspondence and Correlation Analysis

Correspondence analysis (CoA), a multivariate statistical approach, was employed to examine relationships among synonymous codon usage patterns (CUP) across viral sequences [33]. In this study, each CCV coding sequence was represented in 59 dimensions (excluding the termination codons (TAA, TAG, TGA), the initiation codon (ATG), and the tryptophan codon (TGG)). Each dimension corresponded to the RSCU value of one codon, directly reflecting trends in variation [34]. CoA was performed via CodonW 1.4.2, and the values of axes 1 and 2 (the two factors that contribute the most to CUB) were plotted with the online tool (https://www.chiplot.online/) (accessed on 30 July 2026) for visualization and analysis. Each point represents one sequence.

To explore the influence of mutational and selective pressures on CCV’s CUP, correlation analysis was performed with Origin 2022 (version 9.90.225, OriginLab, Northampton, MA, USA) [2] for the base profile (A, T, C, G, GC), nucleotide composition at the third codon position (A3s, T3s, C3s, G3s, GC3s), ENC, CAI, protein grand average of hydropathy (Gravy) and aromaticity (Aromo) that were calculated by CodonW 1.4.2 [35]. Pearson correlation tests were conducted with *p*-value thresholds set at 0.05 and 0.01.

## 3. Results

### 3.1. Base Profile

To gain an initial understanding of CCV’s CUB, the nucleotide composition of coding sequences was analyzed. In CCV’s complete coding sequences (CCDs), the GC content was 50.31% ± 0.47 (mean ± SD). The nucleotide composition at the third codon position of synonymous codons was A3s (31.85% ± 1.78), T3s (31.99% ± 0.91), C3s (29.40% ± 0.84), and G3s (31.96% ± 1.53), showing a nearly equal distribution. The third-position GC content (GC3s) was 48.34% ± 1.22. For Rep genes, The GC and GC3s contents were 53.14% ± 0.54 and 55.61% ± 1.33, respectively. Among the synonymous codons of CCV’s Rep genes, G3s showed the highest frequency (40.15% ± 1.41), followed by T3s (36.88% ± 1.95), C3s (31.96% ± 1.64), and A3s with the lowest occurrence (17.41% ± 1.78). For Cap genes, the overall GC content was 47.14% ± 0.83, while the GC3s was 40.31% ± 2.17. Analysis of third-position nucleotides revealed A3s as the most abundant (47.22% ± 2.81), followed by C3s (26.59% ± 1.58) and T3s (26.58% ± 1.48), with G3s showing the lowest frequency (23.33% ± 2.36). These results indicated that Rep tended to avoid using codons ending with A, while Cap preferred using codons ending with A.

### 3.2. RSCU Analysis

**Figure 1:**
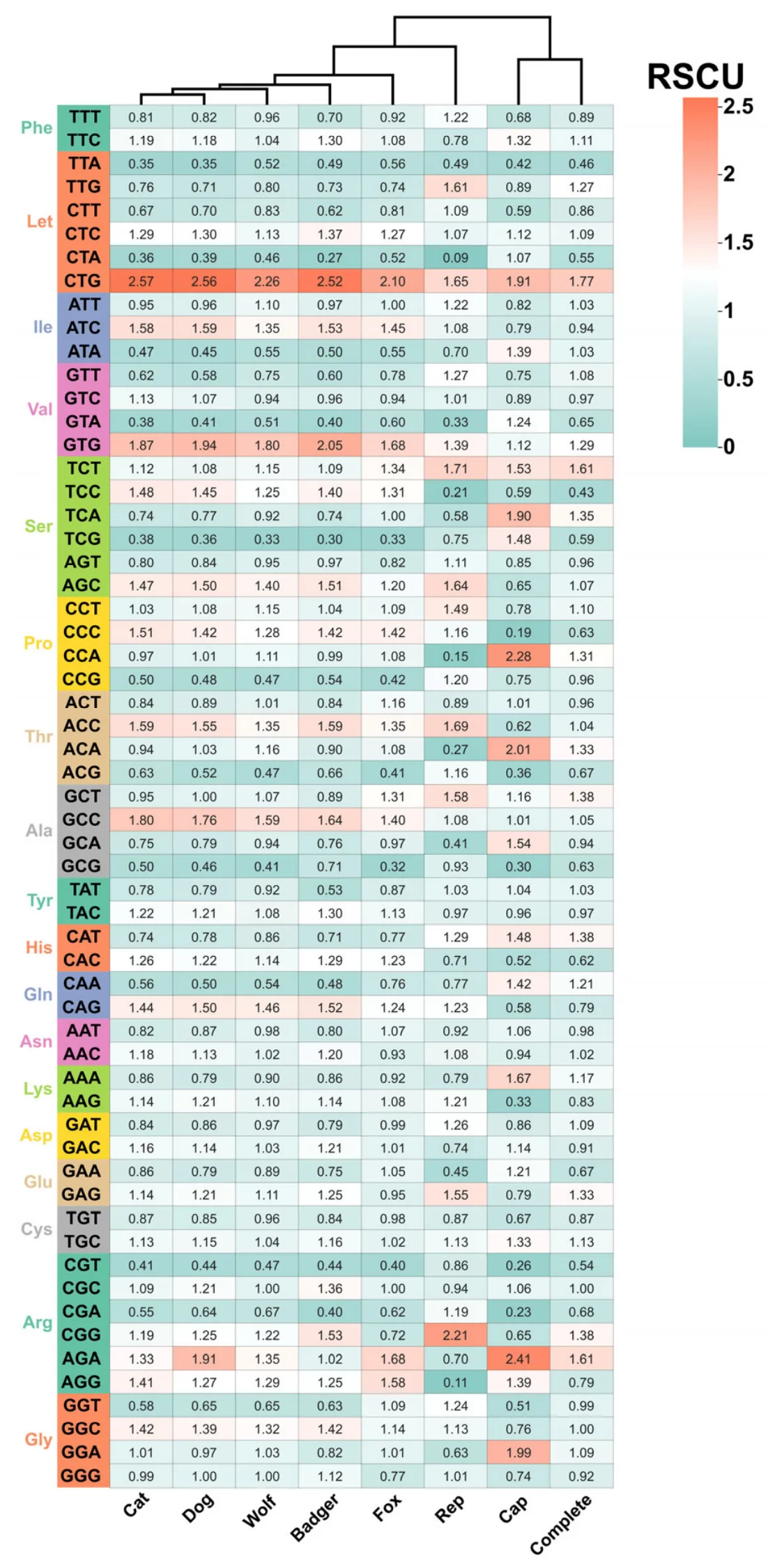
The RSCU heatmap for complete coding sequence (CCD)s, capsid protein (Cap) gene, and the replication protein (Rep) [1] gene of CCV from five different hosts (dog, wolf, fox, cat, and badger). Clustering analysis is shown at the top of the heatmap.

RSCU indices were derived for both CCV and its host species to compare their CUP and assess adaptation levels. As shown in Figure 1, 66.67% (12/18) of the preferred codons for the CCDs end with A/T, which is consistent with the Rep gene (50%, 9 out of 18) and the Cap gene (77.78%, 14 out of 18). Fifty percent (9 of 18) of the optimal codons of Rep end in T, 3 in C, and 6 in G. In Cap, 61.11% (11 of 18) of the most preferred codons end in A, 3 end in T, 2 end in G, and 2 end in C. Rep contains 6 overrepresented codons (RSCU > 1.6) and 9 underrepresented codons (RSCU < 0.6) while Cap contains 7 overrepresented and 12 underrepresented codons (Supplementary Table S2). Notably, none of the 18 optimal codons in the Rep gene end with A, and 7 of the 9 underrepresented codons end in A. These results demonstrate a strong avoidance of codons ending with A in the Rep gene, whereas the Cap gene prefers codons ending with A. A comparison of the synonymous codon usage between CCVs and hosts indicates that most codons are used equally in both CCV and the hosts. For the CCDs, 88.14% (52 out of 59) of the codons are used equally in foxes. Similar patterns are observed in wolves (83.05%, 49 out of 59), dogs (81.35%, 48 out of 59), badgers (81.35%, 48 out of 59), and cats (79.66%, 47 out of 59). Based on the RSCU cluster analysis, we infer that foxes are most similar to CCVs in their Rep synonymous codon usage, followed by badgers, wolves, dogs, and cats.

### 3.3. CAI Analysis

**Figure 2:**
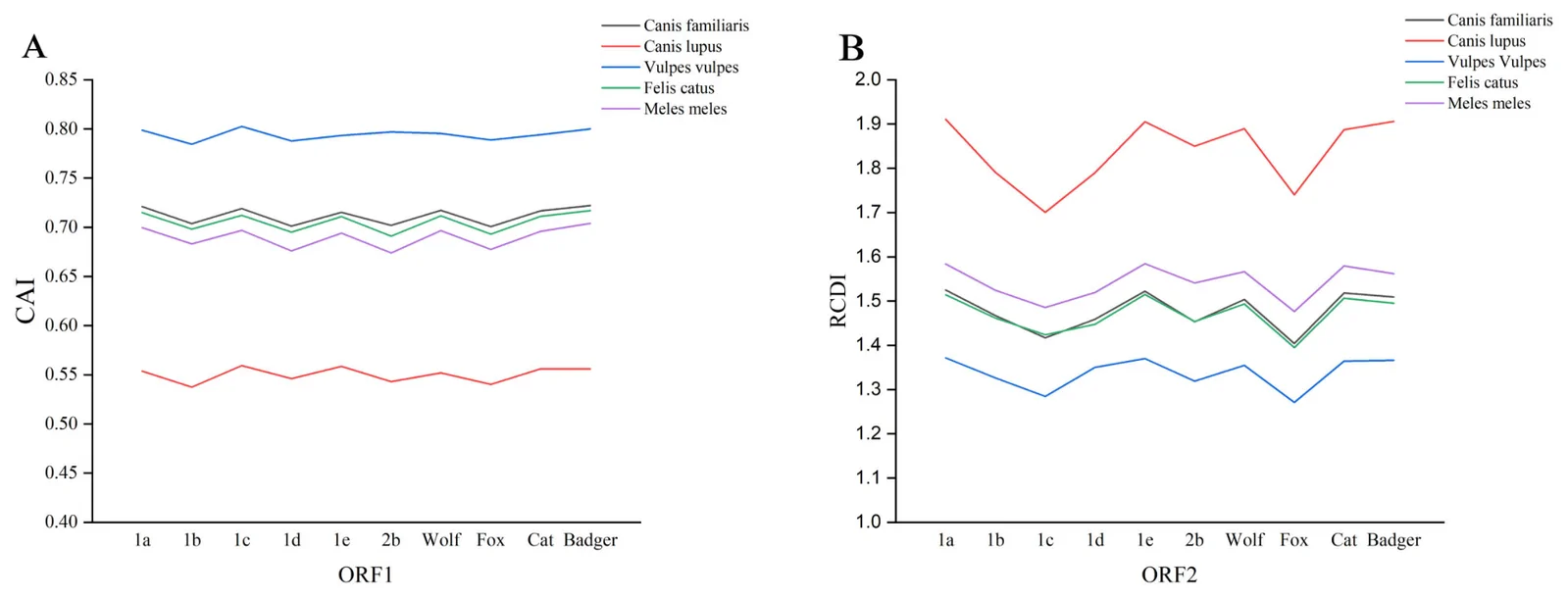
The 102 sequences were classified into 10 groups based on genotype and isolation host. The mean CAI and RCDI values for each host are shown in different colors in the figure. (**A**) Codon adaptation index (CAI) [12] values of ORF1 [1] gene sequences from CCV strains across different genotypes and hosts. (**B**) RCDI values of CCV ORF2 (Cap) gene sequences across different genotypes and hosts (genotypes 1a, 1b, 1c, 1d, 1e, and 2b were obtained from previous genotyping of CCV isolated from dogs).

CAI and RCDI were calculated to assess the adaptation of CCV to different hosts, as shown in Figure 2. The mean CAI values for the Rep and Cap genes were 0.698 ± 0.07 and 0.701 ± 0.04, respectively, with no significant difference at the 95% confidence level (*p* > 0.05). Among different hosts, the highest mean CAI value for the Rep gene was found in *Vulpes vulpes* (fox) (0.794 ± 0.0055), followed by *Canis familiaris* (dog) (0.712 ± 0.0084), *Felis catus* (cat) (0.706 ± 0.0094), and *Meles meles* (badger) (0.690 ± 0.0104). The lowest value was found in *Canis lupus* (wolf) [6] (0.550 ± 0.0075). The lowest RCDI value was found in *Vulpes vulpes* (fox) (1.338 ± 0.0345), followed by *Canis familiaris* (dog) (1.448 ± 0.0421), *Felis catus* (cat) (1.471 ± 0.0388), and *Meles meles* (badger) (1.542 ± 0.0379). The highest value was found in *Canis lupus* (wolf) [6] (1.837 ± 0.0726). Based on RCDI theory, it can be speculated that CCV genes are translated more efficiently in foxes than in the other four hosts and are therefore more suitable for foxes.

**Figure 3:**
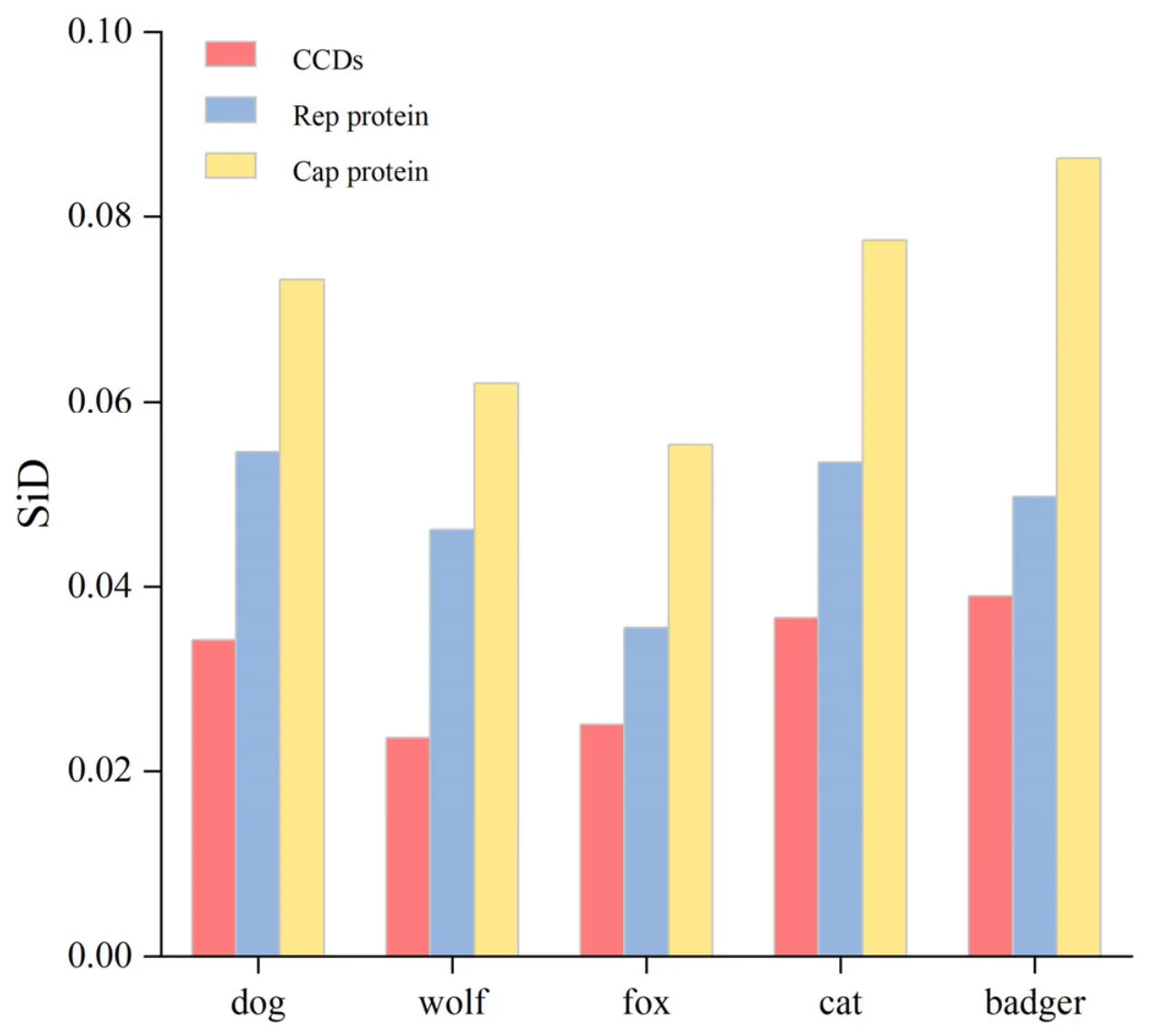
Similarity index for CCV’s complete coding sequences (CCDs), ORF1 (Rep) [1], and ORF2 (Cap) coding sequences in relation to various hosts (dog, wolf, fox, cat, and badger).

Additionally, the similarity index [26] was computed to evaluate the host CUPs’ impact on the virus. As shown in Figure 3, foxes had the lowest SiD values for Rep and Cap among the different hosts. These lower SiD values suggested that CCV codon usage was more similar to that of foxes, possibly suggesting stronger adaptation to foxes. In addition, the SiD value of Cap gene was greater than that of the Rep gene, a pattern that was consistent across different hosts, indicating that host codon usage patterns had a greater effect on codon bias in the coding region of Cap than in that of Rep.

### 3.4. Effect of Selective and Mutation Pressure on Codon Usage Patterns

**Figure 4:**
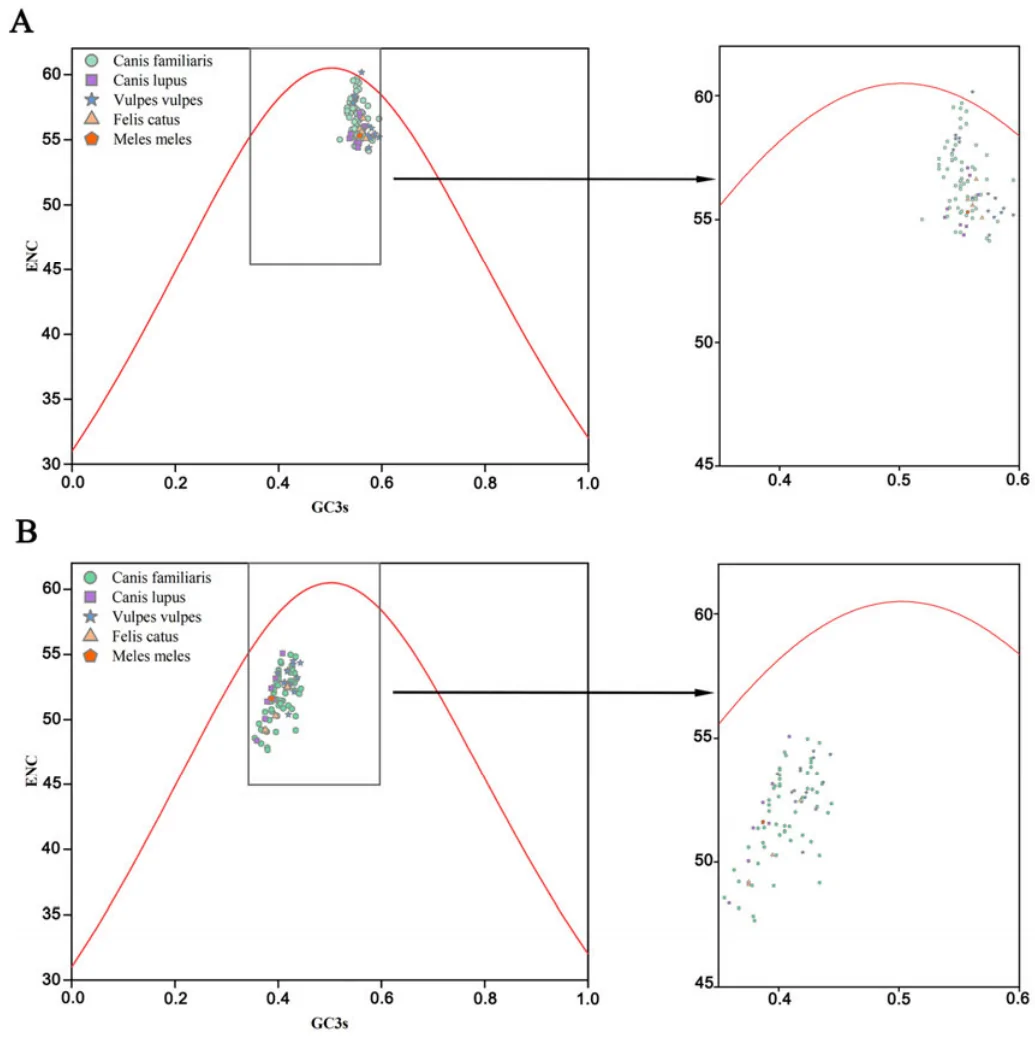
The ENC plot shows the relationship between ENC values and GC3s for the two ORFs, with panel (**A**) corresponding to Rep and panel (**B**) to Cap.

ENC was analyzed to evaluate the CUB levels in CCV and showed significant differences among CCV CCDs from different hosts (*p* < 0.05). The mean ENC value for all CCV CCDs was 58.68 ± 1.49. The mean ENC value for the Rep gene (56.5 ± 1.40) was higher than that for the Cap gene (51.89 ± 1.77), indicating that the Cap gene has a stronger CUB than the Rep gene. Plotting the ENC values against GC3s content revealed that some points for the Rep gene were near the expected curve (mainly from dogs), but most were situated below it (Figure 4A). Meanwhile, all points for the Cap gene were below the predicted curve (Figure 4B). These results indicated that, in addition to mutational pressure, natural selection contributed to shaping the codon usage bias (CUB) of CCV. Furthermore, the point representing the Cap gene was farther away from the standard curve than the point representing the Rep gene, indicating a greater impact of natural selection on the CUB of Cap than on that of Rep.

**Figure 5:**
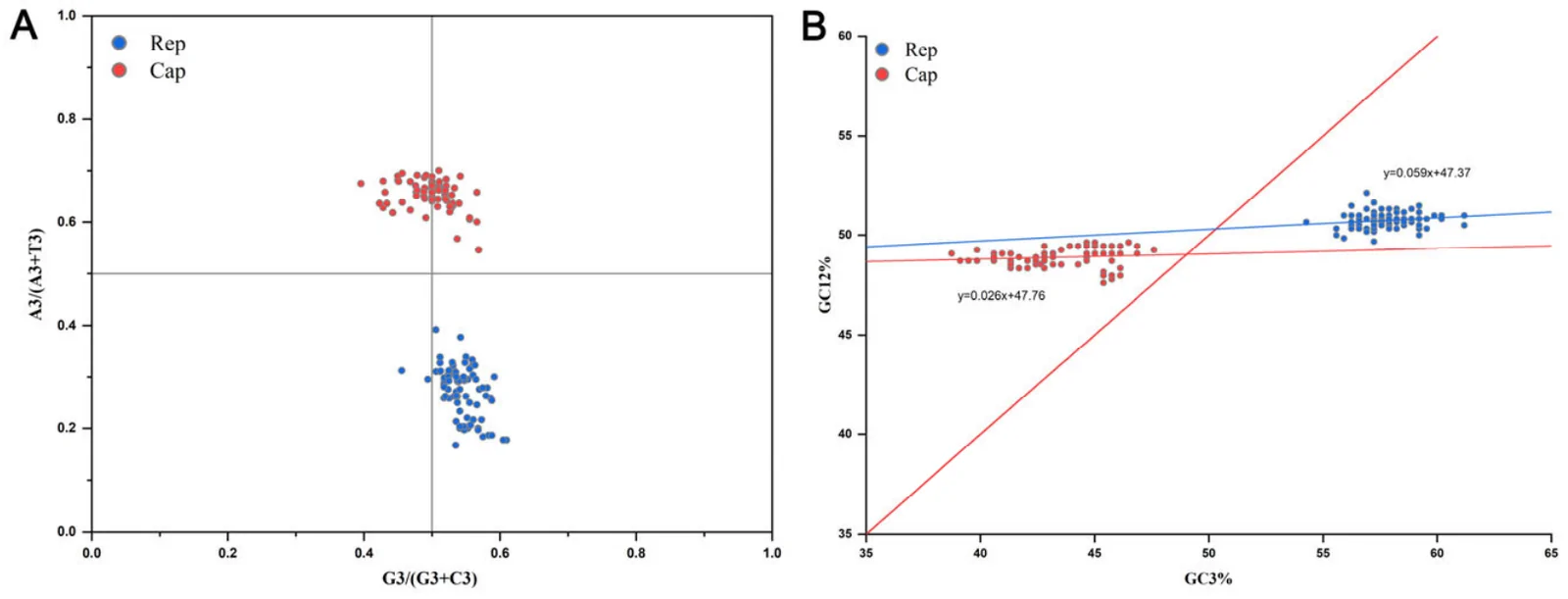
(**A**) PR2 plot for ORF1 (Rep) [1] and ORF2 (Cap). The x-axis represents the G_3_/(G_3_ + C_3_) ratio, and the y-axis represents the A_3_/(A_3_ + T_3_) ratio. (**B**) Neutrality plot analysis showing the correlation between GC12% and GC3% in ORF1 (Rep protein) and ORF2 (Cap protein).

In the parity rule 2 (PR2) plot analysis, central clustering of points would indicate mutation pressure as the predominant force shaping CUB. However, the observed asymmetric distribution across quadrants suggests additional influences from natural selection and/or compositional constraints. As shown in Figure 5, the points for the Rep gene were primarily in the region with G3/(G3 + C3) > 0.5 and A3/(A3 + T3) < 0.5, indicating greater use of G/T than of C/A at the third codon position. For the Cap gene, the points indicated A > T, while G and C were relatively balanced. These findings aligned with the RSCU analysis, confirming that CCV’s CUB was affected by both natural selection and mutational pressure.

A previous study has demonstrated that mutational pressure predominantly affects GC12, whereas natural selection primarily influences third-position GC content (GC3) [13]. To investigate the predominant factors shaping CUB in CCVs, neutrality plot analysis was performed by fitting GC12 to GC3 linearly. As shown in Figure 5, the slope of the fitted curve for Rep gene was 0.026 (R^2^ = 0.016, *p* > 0.05), and that for the Cap gene was 0.059 (R^2^ = 0.030, *p* > 0.05). The results showed that GC3 had a higher rate of change than GC12, indicating that natural selection contributed 97.4% to CUB for Rep, which was greater than that of 94.1% for Cap. The linear correlations between GC12 and GC3 in Rep and Cap were not significant, indicating that GC12 content operates independently of GC3 in both viral genes. These findings suggest that natural selection may contribute to shaping the codon usage bias of CCV, but the low R^2^ values and non-significant regressions indicate that precise quantitative attribution to selection versus mutation is not reliable.

### 3.5. Correspondence and Correlation Analysis

**Figure 6:**
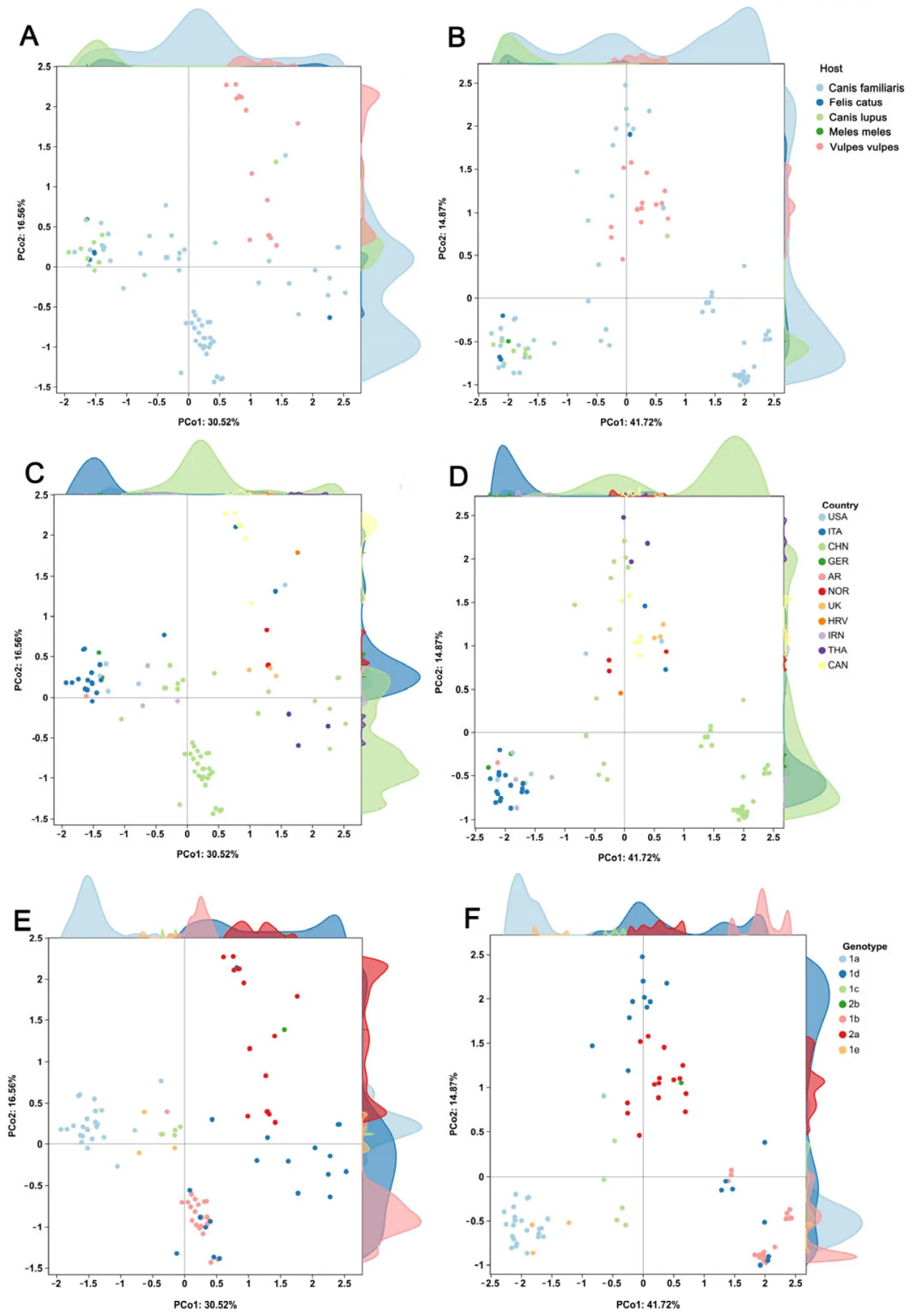
RSCU-based correspondence analysis (CoA) stratified by host (**A**,**B**), geographic factors (**C**,**D**), and genotype (**E**,**F**) across the Rep and Cap CDS of CCV. The predominant factor (axis 1) accounts for 30.52% and 41.72% of the overall variation, while the secondary factor (axis 2) accounts for 16.56% and 14.87% of the overall variation in the Rep and Cap CDS, respectively.

To explore variation in synonymous CUP of CCV, a correspondence analysis (CoA) was performed on the RSCU scores of the Rep and Cap genes. The distribution along predominant and secondary factors is shown in Figure 6. For the Rep gene, the predominant and secondary factors account for 30.52% and 16.56% of the total variance, respectively. For the Cap gene, they account for 41.72% and 14.87% of the total variance, respectively. The CoA analysis suggested that formation of CCV’s codon preference was multifactorial. For both Rep and Cap genes, strains from foxes were clustered separately in the first and second quadrants, with a codon usage pattern (CUP) different from that of other strains. Strains from wolves, cats, and some strains from dogs were clustered together in the second and third quadrants. These results indicated that the hosts might influence the relative synonymous CUP of CCV (Figure 6A,B). In addition, CoA of CCVs from different countries was also performed. The results showed that strains from Iran, the US, and Italy were clustered together, while some strains from China (mainly the CCV-1b genotype) clustered separately (Figure 6C–F). For the Rep and Cap genes, CCV-2a and 2b were clustered together; 1a,1e,1c were clustered together; and 1b and some 1d strains were clustered together [17].

Furthermore, we also performed correlation analysis to comprehensively assess the impact of selective pressure and mutational forces on CCV’s CUB. For the Rep gene, ENC and CAI were highly significantly (*p* < 0.01) correlated with Gravy. For the Cap gene, ENC was significantly correlated (*p* < 0.05) with Gravy and highly significantly correlated with Aromo. CAI was highly significantly correlated with Gravy. Further analysis indicated that, the predominant factor for the Rep gene was highly significantly correlated with CAI; for the Cap gene, the secondary factor was significantly or highly significantly correlated with CAI, Gravy, and Aromo. These results showed that the properties of amino acids and host translational selection affected the CUB of CCV, supporting a role for natural selection.

**Figure 7:**
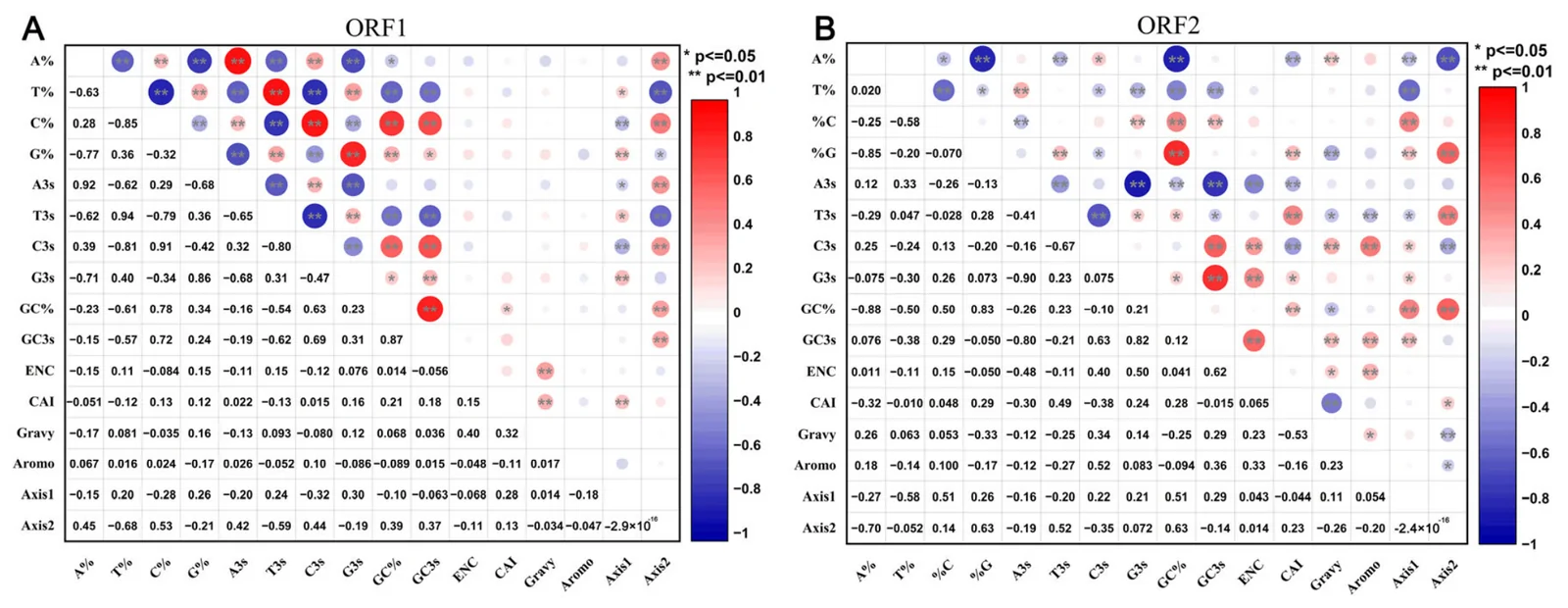
Correlation analysis among various metrics in CCV Cap (**A**) versus Rep (**B**). Dark blue denotes negative correlation, whereas dark red indicates positive correlation. Higher values denote stronger correlations and the bigger dot. Asterisks “*” and “**” indicate statistical significance at *p* ≤ 0.05 and *p* ≤ 0.01, respectively.

The correlations among the base profile (A%, T%, C%, G%, GC%), nucleotide composition at the third position of synonymous codons (A3s, T3s, C3s, G3s, GC3s), and ENC were analyzed. As shown in Figure 7, for Rep and Cap, most third-position nucleotide compositions were significantly or highly significantly correlated with nucleotide compositions, indicating that compositional constraints could affect synonymous codon usage preferences. No significant correlation was found between ENC and base composition, demonstrating that base composition was not the predominant factor affecting CCV’s CUB. Further correlation analyses showed that the predominant and secondary factors had significant or highly significant correlations with nucleotide composition in both Rep and Cap, suggesting that nucleotide composition affected the CUB of CCV.

## 4. Discussion

Canine circovirus (CCV) is found mostly in dogs and could cause immunosuppression, making them more susceptible to other viruses. CCV also has a strong ability to cross species barriers, posing a serious threat to the health of other carnivores (wolves, foxes, cats, and badgers). Although many studies have been conducted on the epidemiology, genetic recombination, phylogenetic and evolutionary origins of CCV, its pathogenic mechanism remains unclear. Moreover, CCV cannot be cultured in cells, which limits understanding of the relationship between the virus and its host during infection [6,36].

Codon usage bias (CUB) analysis could help elucidate evolutionary processes and viral adaption to the host [23]. This study revealed that CCV’s complete coding sequences (CCDs) exhibit a moderate GC content (50.31%) and a high ENC value (58.68), reflecting weak CUB. These characteristics align with those of avian circoviruses and parvoviruses, including duck circovirus (GC content = 50.70%, ENC = 59.34), goose astrovirus (GC content = 41.33%, ENC = 52.2), goose circovirus (GC content = 49.01%, ENC = 59.20), and goose parvovirus (GC content = 44.60%, ENC = 50.01). In contrast, other viruses exhibiting stronger CUB had extreme GC content and lower ENC, such as canine parvovirus (GC content = 36.25%, ENC = 36.12) and bovine herpesvirus-1 (GC content = 72.35%, ENC = 36.59), suggesting that mutational pressure represented the principal force shaping codon usage. Comparative analysis demonstrated significantly higher ENC values for the Rep gene than those for the Cap gene (Figure 4), indicating that the Cap gene might have stronger CUB than the Rep gene. The Cap protein is associated with host immune responses, and higher CUB implies preferential use of specific codon combinations to increase translational efficiency and stability. Notably, our analysis revealed a strong preference for A-ending synonymous codons in the CCV-Cap coding sequence and a strong avoidance of T-ending synonymous codons in the Rep coding sequence, which is consistent with the results for most circular single-stranded DNA viruses, such as goose circovirus and porcine circovirus [37,38]. The observed A-ending codon preference in the CCV-Cap gene might optimize viral survival and replication, while the overall weak CUB (ENC > 50) could facilitate cross-species transmission by reducing host-specific translational constraints [26,39]. Earlier studies have demonstrated that the preference for A-ending codons suggests mutational pressure as the major factor shaping viral CUB [26]. However, our analysis showed that natural selection was the major determinant in CCV’s CUB, indicating that other regulatory mechanisms underlying CCV codon usage remain to be investigated.

Comparative analysis revealed the highest codon usage pattern (CUP) similarity between CCV and foxes (88.14% matched codons in complete coding sequences). Gene-specific analysis showed greater codon usage alignment between foxes and Cap (69.49% matched codons) than between foxes and Rep (66.10%), suggesting differential codon usage between viral genes. Studies have shown that similar CUP between a virus and its host may enhance the viral extension rate and thus accelerate the translation process, whereas opposite patterns can ensure proper protein folding [40]. Host adaptation analysis suggested that foxes were the most suitable hosts for CCV because they had the highest CAI and the lowest RCDI and SiD values. Meanwhile, RSCU cluster analysis indicated that cats and dogs had the most similar CUP, suggesting that CCV may also be widespread in cats. Keeping cats and dogs together is extremely common in modern cities, which provides favorable conditions for CCV to spread from dogs to cats. The scarcity of reports of CCV in cats might reflect limited investigation of CCV in cats.

Previous studies have shown that CUB was primarily driven by natural selection and mutational pressure [15]. ENC, PR2, and neutrality plot analyses demonstrated that CCV’s CUP resulted from the interplay of natural selection and mutation pressure, with natural selection exerting a significantly greater effect. Significant correlations between ENC and Gravy, as well as Aromo, further validated the crucial role of natural selection for the Cap gene. Notably, the correlation among Rep’s nucleotide composition, third-position nucleotide composition, and ENC was stronger than that for Cap, suggesting that the Rep gene was subject to greater mutational pressure than the Cap gene, which was consistent with the neutrality plot analysis. Generally, higher mutational pressure plays a positive role in driving viral evolution and genetic diversity. Furthermore, the correlation analysis indicated that CUB of CCV was also influenced by compositional constraints and the properties of amino acids. CoA analysis revealed that CCV could be broadly classified into three distinct clusters, and the peak cluster plot demonstrated highly aggregated distributions for strains originating from Germany and those from foxes along the predominant factor. These clustering patterns suggested that both host species and geographical factors might influence CCV’s CUB. However, the complex interplay between these evolutionary forces and their relative contributions to viral adaptation dynamics remain incompletely characterized. One possible explanation is that following its initial emergence in Norway, CCV was introduced to diverse geographical regions via different host species. Over extended evolutionary periods, the combined selective pressures arising from regional climatic/temperature variations and the inherent codon usage preferences of distinct local host populations potentially drove the gradual shaping of the contemporary CUPs observed in CCV [23,41]. In general, genes with stronger codon preferences may have higher translational efficiency. Correlational analysis revealed that ENC and CAI for both Rep (r = 0.15, *p* > 0.05) and Cap (r = 0.065, *p* > 0.05) showed a positive but nonsignificant correlation, suggesting that there might be other unknown mechanisms of expression regulation between CCV and the host. These findings are predictions based on codon usage and require epidemiological and experimental validation. Although all CCV sequences isolated from foxes, cats, and badgers were included, the small sample size remains a limitation of this study. Codon usage bias is influenced not only by the host, but also jointly by geographic origin, genotype, and phylogenetic relatedness. Future work should also incorporate phylogenetic correction based on additional sequences from newly identified strains.

## 5. Conclusions

In conclusion, the analysis indicated that CCV’s CUP was affected by natural selection and mutational pressure, as well as geographic factors, different hosts and genotypes. The Rep protein was subject to greater mutational pressure compared to the Cap protein, while the overall codon preference of CCV was weak. Based on host adaptation analysis, we speculate that CCV was also adapted to hosts other than dogs and cats, such as foxes. These findings could contribute to the study of the cross-species transmission and pathogenic mechanism of CCV and ultimately support the development of effective CCV prevention and control measures.

## Data Availability

All data generated or analyzed during this study are included in this article or the Supplementary Materials (Supplementary Tables S1 and S2). The datasets generated during and/or analyzed during the current study are available in the Figshare repository, (https://doi.org/10.6084/m9.figshare.27710010).

## Supplementary Materials

The following supporting information can be downloaded at: https://www.mdpi.com/article/10.3390/ani16213494/s1, Table S1: The detailed information of reference strains used in this study; Table S2: RSCU of CCV and each host (CCV and the preferred codon usage for 18 amino acids in various hosts are displayed bolded).
