# Supplementary material for: Codon Usage Bias and Host Adaptation of Canine Circovirus Identified from Various Hosts
Source: Animals (Basel). 2026 Nov 4;16(21):3494. doi: 10.3390/ani16213494 (PMC13761561; doi:10.3390/ani16213494)
Supplement: Supplementary file 1 [file animals-16-03494-s001.zip › animals-4589195-supplementary.pdf]

Supplementary Table S1. The detailed information of reference strains used in this study

Strains	Sample	Source	Date	Country	Accession
**214**	serum	*Canine familiaris*	2011	USA	JQ821392.1
**ITA/2021/cat/99.95**	serum	*Felis catus*	2021	Italy	OQ357581.1
**ITA/2021/cat/230.1**	serum	*Felis catus*	2021	Italy	OQ357582.1
**ITA/2021/cat/230.3**	serum	*Felis catus*	2021	Italy	OQ357583.1
**GZ-9/2022**	nasal swabs	*Felis catus*	2022	China	OP575982.1
**XF16**	feces	*Canine familiaris*	2016	China	MF797786.1
**K1**	feces	*Canine familiaris*	2016	China	MK731982.1
**C24**	feces	*Canine familiaris*	2016	China	MK731981.1
**C79**	feces	*Canine familiaris*	2016	China	MK944079.1
**Ha13**	EDTA blood	*Canine familiaris*	2013	Germany	KF887949.1
**JZ98/2014**	N.A.	*Canine familiaris*	2014	China	KT946839.1
**CD17/2016**	N.A.	*Canine familiaris*	2016	China	MG266899.1
**PE8575/1-13**	intestine	*Canine familiaris*	2013	Italy	KT734823.1
**TE7482-13**	intestine	*Canine lupus*	2013	Italy	KT734822.1
**AZ663/2-13**	intestine	*Canine lupus*	2013	Italy	KT734824.1
**AZ4133/2-13**	intestine	*Canine lupus*	2013	Italy	KT734827.1
**CB6293/2-14**	intestine	*Canine lupus*	2014	Italy	KT734828.1
**TE6685/1-13**	brain	*Canine familiaris*	2013	Italy	KT734821.1
**AZ663/1-13**	spleen	*Canine lupus*	2013	Italy	KT734820.1
**AZ5586-13**	lung	*Canine lupus*	2013	Italy	KT734819.1
**AZ5212/1-14**	liver	*Canine familiaris*	2014	Italy	KT734817.1
**AZ4438-13**	intestine	*Meles meles*	2013	Italy	KT734816.1
**TE4016-13**	spleen	*Canine lupus*	2013	Italy	KT734814.1
**AZ4133/1-13**	spleen	*Canine lupus*	2013	Italy	KT734815.1
**AZ2972-13**	intestine	*Canine familiaris*	2013	Italy	KT734813.1
**CB6293/1-14**	mesenteric lymph nodes	*Canine lupus*	2014	Italy	KT734812.1
**CB6293/1-14**	mesenteric	*Canine familiaris*	2014	Germany	KT283604.1

	lymph nodes				
**UCD1-1698**	liver tissue	*Canine familiaris*	2011	USA	KC241982.1
**UCD2-32162**	feces	*Canine familiaris*	2011	USA	KC241984.1
**UCD3-478**	blood	*Canine familiaris*	2011	USA	KC241983.1
**204**	N.A.	*Canine familiaris*	2017	China	MG279141.1
**205**	N.A.	*Canine familiaris*	2017	China	MG279140.1
**384**	N.A.	*Canine familiaris*	2017	China	MG279139.1
**388**	N.A.	*Canine familiaris*	2017	China	MG279138.1
**390**	N.A.	*Canine familiaris*	2017	China	MG279137.1
**391**	N.A.	*Canine familiaris*	2017	China	MG279136.1
**201**	N.A.	*Canine familiaris*	2017	China	MG279135.1
**394**	N.A.	*Canine familiaris*	2017	China	MG279134.1
**395**	N.A.	*Canine familiaris*	2017	China	MG279133.1
**202**	N.A.	*Canine familiaris*	2017	China	MG279131.1
**177**	N.A.	*Canine familiaris*	2017	China	MG279130.1
**178**	N.A.	*Canine familiaris*	2017	China	MG279129.1
**179**	N.A.	*Canine familiaris*	2017	China	MG279128.1
**180**	N.A.	*Canine familiaris*	2017	China	MG279127.1
**181**	N.A.	*Canine familiaris*	2017	China	MG279126.1
**182**	N.A.	*Canine familiaris*	2017	China	MG279125.1
**183**	N.A.	*Canine familiaris*	2017	China	MG279124.1
**OH19098-1**	N.A.	*Canine familiaris*	2015	USA	MF457592.1
**398**	N.A.	*Canine familiaris*	2017	China	MG279132.1
**UBA-Baires**	viscus	*Canine familiaris*	2018	Argentina	MK033608.1
**WM72**	N.A.	*Canine familiaris*	2015	China	KY388503.1
**WM74**	N.A.	*Canine familiaris*	2015	China	KY388502.1
**WM76**	N.A.	*Canine familiaris*	2015	China	KY388501.1
**WM77**	N.A.	*Canine familiaris*	2015	China	KY388500.1
**WM79**	N.A.	*Canine familiaris*	2015	China	KY388499.1
**WM83**	N.A.	*Canine familiaris*	2015	China	KY388498.1

**WM84**	N.A.	*Canine familiaris*	2015	China	KY388497.1
**XXT242**	N.A.	*Canine familiaris*	2015	China	KY388496.1
**XXT243**	N.A.	*Canine familiaris*	2015	China	KY388495.1
**YL11**	N.A.	*Canine familiaris*	2015	China	KY388494.1
**WM62**	N.A.	*Canine familiaris*	2015	China	KY388493.1
**WM66**	N.A.	*Canine familiaris*	2015	China	KY388492.1
**WM63**	N.A.	*Canine familiaris*	2015	China	KY388491.1
**JZ82**	N.A.	*Canine familiaris*	2015	China	KY388490.1
**JZ85**	N.A.	*Canine familiaris*	2014	China	KY388489.1
**LA128**	N.A.	*Canine familiaris*	2014	China	KY388488.1
**LA237**	N.A.	*Canine familiaris*	2014	China	KY388487.1
**LA280**	N.A.	*Canine familiaris*	2014	China	KY388486.1
**WM46**	N.A.	*Canine familiaris*	2015	China	KY388485.1
**WM48**	N.A.	*Canine familiaris*	2015	China	KY388484.1
**WM60**	N.A.	*Canine familiaris*	2015	China	KY388483.1
**GL51**	N.A.	*Canine familiaris*	2015	China	KY388482.1
**GL33**	N.A.	*Canine familiaris*	2015	China	KY388480.1
**CanineCV-447/2017**	spleen	*Canine lupus*	2017	Italy	MW829201.1
**CanineCV-448/2017**	spleen	*Canine lupus*	2017	Italy	MW829202.1
**CanineCV-449/2017**	spleen	*Canine lupus*	2017	Italy	MW829203.1
**CanineCV-450/2017**	intestine	*Canine lupus*	2017	Italy	MW829204.1
**CanineCV-454/2018**	intestine	*Canine lupus*	2018	Italy	MW829205.1
**CanineCV-457/2018**	intestine	*Canine lupus*	2018	Italy	MW829206.1
**CanineCV-458/2018**	intestine	*Canine lupus*	2018	Italy	MW829207.1
**Bari/411-13**	intestine	*Canine familiaris*	2013	Italy	KJ530972.1
**27/2014**	spleen	*Vulpes vulpes*	2014	Norway	MT180079.1
**02/1996**	liver	*Vulpes vulpes*	1996	Norway	MT180083.1
**55/1998**	spleen	*Vulpes vulpes*	1998	Norway	MT180086.1
**VS7100001**	Serum	*Vulpes vulpes*	2013	UK	KP260925.1
**VS7100003**	Serum	*Vulpes vulpes*	2013	UK	KP260926.1

**VS7100005**	Serum	*Vulpes vulpes*	2013	UK	KP260927.1
**55590**	feces	*Vulpes vulpes*	2014	Croatia	KP941114.1
**IRN/2019/Dog/292**	rectal swabs	*Canine familiaris*	2019	Iran	OK625288.1
**IRN/2019/Dog/298**	rectal swabs	*Canine familiaris*	2019	Iran	OK625289.1
**IRN/2019/Dog/498**	rectal swabs	*Canine familiaris*	2019	Iran	OK625293.1
**WD-R025/TH2020**	nasal/oropharyngeal swabs	*Canine familiaris*	2020	Thailand	MZ826144.1
**WD-H026/TH2020**	nasal/oropharyngeal swabs	*Canine familiaris*	2020	Thailand	MZ826147.1
**WD-H070/TH2020**	nasal/oropharyngeal swabs	*Canine familiaris*	2020	Thailand	MZ826148.1
**NP28**	spleen	*Vulpes vulpes*	2022	Canada	ON418893.1
**FNA8**	spleen	*Vulpes vulpes*	2022	Canada	ON418896.1
**FNA7**	spleen	*Vulpes vulpes*	2022	Canada	ON418897.1
**FNA4**	spleen	*Vulpes vulpes*	2022	Canada	ON418898.1
**FLC30**	spleen	*Vulpes vulpes*	2022	Canada	ON418899.1
**FLC27**	spleen	*Vulpes vulpes*	2022	Canada	ON418900.1
**FGB7**	spleen	*Vulpes vulpes*	2022	Canada	ON418904.1
**FGB6**	spleen	*Vulpes vulpes*	2022	Canada	ON418905.1

Supplementary Table S2. RSCU of CCV and each host (CCV and the preferred codon usage for 18 amino acids in various hosts are displayed bolded).

		RSCU							
**AA**	**Condon**	**Complete**	**Rep**	**Cap**	**Dog**	**Wolf**	**Fox**	**Cat**	**Badger**
**F**	TTT	0.89	**1.22**	0.68	0.82	0.96	0.92	0.81	0.70
	TTC	**1.11**	0.78	**1.32**	**1.18**	**1.04**	**1.08**	**1.19**	**1.30**
**L**	TTA	0.46	0.49	0.42	0.35	0.52	0.56	0.35	0.49
	TTG	1.27	1.61	0.89	0.71	0.80	0.74	0.76	0.73
	CTT	0.86	1.09	0.59	0.70	0.83	0.81	0.67	0.62
	CTC	1.09	1.07	1.12	1.30	1.13	1.27	1.29	1.37
	CTA	0.55	0.09	1.07	0.39	0.46	0.52	0.36	0.27
	CTG	**1.77**	**1.65**	**1.91**	**2.56**	**2.26**	**2.10**	**2.57**	**2.52**
**I**	ATT	**1.03**	**1.22**	0.82	0.96	1.10	1.00	0.95	0.97
	ATC	0.94	1.08	0.79	**1.59**	**1.35**	**1.45**	**1.58**	**1.53**
	ATA	**1.03**	0.70	**1.39**	0.45	0.55	0.55	0.47	0.50

**V**	GTT	1.08	1.27	0.75	0.58	0.75	0.78	0.62	0.60
	GTC	0.97	1.01	0.89	1.07	0.94	0.94	1.13	0.96
	GTA	0.65	0.33	**1.24**	0.41	0.51	0.60	0.38	0.40
	GTG	**1.29**	**1.39**	1.12	**1.94**	**1.80**	**1.68**	**1.87**	**2.05**
**S**	TCT	**1.61**	**1.71**	1.53	1.08	1.15	**1.34**	1.12	1.09
	TCC	0.43	0.21	0.59	1.45	1.25	1.31	**1.48**	1.40
	TCA	1.35	0.58	**1.90**	0.77	0.92	1.00	0.74	0.74
	TCG	0.59	0.75	1.48	0.36	0.33	0.33	0.38	0.30
	AGT	0.96	1.11	0.85	0.84	0.95	0.82	0.80	0.97
	AGC	1.07	1.64	0.65	**1.50**	**1.40**	1.20	1.47	**1.51**
**P**	CCT	1.10	**1.49**	0.78	1.08	1.15	1.09	1.03	1.04
	CCC	0.63	1.16	0.19	**1.42**	**1.28**	**1.42**	**1.51**	**1.42**
	CCA	**1.31**	0.15	**2.28**	1.01	1.11	1.08	0.97	0.99
	CCG	0.96	1.20	0.75	0.48	0.47	0.42	0.50	0.54
**T**	ACT	0.96	0.89	1.01	0.89	1.01	1.16	0.84	0.84
	ACC	1.04	**1.69**	0.62	**1.55**	**1.35**	**1.35**	**1.59**	**1.59**
	ACA	**1.33**	0.27	**2.01**	1.03	1.16	1.08	0.94	0.90
	ACG	0.67	1.16	0.36	0.52	0.47	0.41	0.63	0.66
**A**	GCT	**1.38**	**1.58**	1.16	1.00	1.07	1.31	0.95	0.89
	GCC	1.05	1.08	1.01	**1.76**	**1.59**	**1.40**	**1.80**	**1.64**
	GCA	0.94	0.41	**1.54**	0.79	0.94	0.97	0.75	0.76
	GCG	0.63	0.93	0.30	0.46	0.41	0.32	0.50	0.71
**Y**	TAT	**1.03**	**1.03**	**1.04**	0.79	0.92	0.87	0.78	0.53
	TAC	0.97	0.97	0.96	**1.21**	**1.08**	**1.13**	**1.22**	**1.30**
**H**	CAT	**1.38**	**1.29**	**1.48**	0.78	0.86	0.77	0.74	0.71
	CAC	0.62	0.71	0.52	**1.22**	**1.14**	**1.23**	**1.26**	**1.29**
**Q**	CAA	**1.21**	0.77	**1.42**	0.50	0.54	0.76	0.56	0.48
	CAG	0.79	**1.23**	0.58	**1.50**	**1.46**	**1.24**	**1.44**	**1.52**
**N**	AAT	0.98	0.92	**1.06**	0.87	0.98	**1.07**	0.82	0.80
	AAC	**1.02**	**1.08**	0.94	**1.13**	**1.02**	0.93	**1.18**	**1.20**
**K**	AAA	**1.17**	0.79	**1.67**	0.79	0.90	0.92	0.86	0.86
	AAG	0.83	**1.21**	0.33	**1.21**	**1.10**	**1.08**	**1.14**	**1.14**
**D**	GAT	**1.09**	**1.26**	0.86	0.86	0.97	0.99	0.84	0.79
	GAC	0.91	0.74	**1.14**	**1.14**	**1.03**	**1.01**	**1.16**	**1.21**
**E**	GAA	0.67	0.45	**1.21**	0.79	0.89	**1.05**	0.86	0.75
	GAG	**1.33**	**1.55**	0.79	**1.21**	**1.11**	0.95	**1.14**	**1.25**
**C**	TGT	0.87	0.87	0.67	0.85	0.96	0.98	0.87	0.84
	TGC	**1.13**	**1.13**	**1.33**	**1.15**	**1.04**	**1.02**	**1.13**	**1.16**
**R**	CGT	0.54	0.86	0.26	0.44	0.47	0.40	0.41	0.44
	CGC	1.00	0.94	1.06	1.21	1.00	1.00	1.09	1.36
	CGA	0.68	1.19	0.23	0.64	0.67	0.62	0.55	0.40
	CGG	1.38	**2.21**	0.65	1.25	1.22	0.72	1.19	**1.53**
	AGA	**1.61**	0.70	**2.41**	**1.91**	**1.35**	**1.68**	1.33	1.02
	AGG	0.79	0.11	1.39	1.27	1.29	1.58	**1.41**	1.25
**G**	GGT	0.99	**1.24**	0.51	0.65	0.65	1.09	0.58	0.63
	GGC	1.00	1.13	0.76	**1.39**	**1.32**	**1.14**	**1.42**	**1.42**
	GGA	**1.09**	0.63	**1.99**	0.97	1.03	1.01	1.01	0.82
	GGG	0.92	1.01	0.74	1.00	1.00	0.77	0.99	1.12
